# SARS-CoV-2: the many pros of targeting PLpro

**DOI:** 10.1038/s41392-020-00335-z

**Published:** 2020-10-06

**Authors:** Christopher B. McClain, Nicolas Vabret

**Affiliations:** 1 Department of Medicine, Hematology and Medical Oncology, Icahn School of Medicine at Mount Sinai, New York, NY USA; 2 Precision Immunology Institute, Icahn School of Medicine at Mount Sinai, New York, NY USA

**Keywords:** Microbiology, Innate immunity, Drug development

A new study^1^ published in *Nature* compares the mechanisms used by SARS-CoV-1 and SARS-CoV-2 papain-like protease (PLpro) to promote innate immune evasion. Its findings identify SARS-CoV-2 PLpro as an attractive drug target for treating COVID-19.

SARS-CoV-2 is a novel human coronavirus (CoV) responsible for the COVID-19 pandemics. The pathogenesis of SARS-CoV-2 is characterized by a strong inhibition of innate immune sensing and production of type I interferon (IFN-I) responses.^2^ SARS-CoV-2 shares 79% of its genome with SARS-CoV-1, the agent responsible of the 2003 SARS epidemics, allowing comparative analyses that can address the molecular determinants in pathogenicity.

In this context, a study by Shin et al.^1^ aimed at identifying similarities and key differences in the activities of PLpro produced by SARS-CoV-1 (SCoV1-PLpro) and by SARS-CoV-2 (SCoV2-PLpro). Encoded by nsp3, PLpro is one of two known CoV proteases and is required for the efficient cleavage of nsp1, nsp2, and nsp3 from the viral polyprotein, a process essential for viral genome transcription and replication.^2,3^ In addition to its role as a viral protease, PLpro from SARS-CoV-1 antagonizes cellular ubiquitination and ISGylation.^4^ Ubiquitination is a post-translational modification characterized by the addition of ubiquitin (Ub) chains to lysine residues of a protein, which regulates its activity, notably via its targeting to proteasomal degradation. ISGylation is a process similar to ubiquitination, where Interferon Stimulated Gene 15 (ISG15), a small protein highly induced by IFN-I, is conjugated to target proteins and modulates their functions. Both ubiquitination and ISGylation play important roles in the regulation of innate immune responses to viral infection, and it may therefore not be surprising to observe that multiple viruses have evolved different strategies to antagonize these pathways.

Shin et al. performed a series of biochemical, molecular, and structural analyses to compare the deISGylating and deubiquitylating activities of SCoV1-PLpro and SCoV2-PLpro. Using ISG15- and K48-linked di-ubiquitin (K48-Ub_2_)-specific activity-based probes, they measured that SCoV2-PLpro was more efficient at cleaving ISG15 than Ub residues in vitro, while observing the opposite result with SCoV1-PLpro. To further understand these contrasting activities, the authors determined the crystal structures of SCoV2-PLpro in complex with murine ISG15 and compared it with SCoV1-PLpro in complex with either murine ISG15 or K48-Ub_2_. The authors first noted that the two PLpros shared the same binding mode to ISG15. However, when comparing with SCoV1-PLpro complexed with K48-Ub_2_, they identified a difference in the S2 helix binding site of the protease: SCoV1-PLpro structure suggested a hydrophobic interaction between Leu76 from PLpro and Ile44 from K48-Ub_2_, while the corresponding interacting residue on SCoV2-PLpro was a polar threonine. Importantly, by mutating SCoV2-PLpro to mirror this hydrophobic interaction, the authors restored a cleavage efficacy on K48-Ub_2_ comparable to the one from SCoV1-PLpro, suggesting the helix S2 binding site is critical for PLpro specificity. The authors further identified additional residues involved in PLpro binding to ISG15 by targeted mutations that resulted in significantly lower enzymatic activity and reactivity towards ISG15. Overall, these results outline a shift in substrate specificity between the PLpros from SARS-CoV-1 and SARS-CoV-2.

To better characterize the consequence of this difference, the authors compared the cellular interactome of catalytically inactive mutants of SCoV1-PLpro and SCoV2-PLpro in a lung cancer cell line stimulated with IFN-I. Confirming their previous findings, ISG15 was found significantly enriched in complexes with PLpro from SARS-CoV-2, while SCoV1-PLpro preferentially bound ubiquitin residues. Further, overexpression of PLpro in IFN-I-treated cells led to inhibition of cellular protein ISGylation, including—and perhaps central to its activity—ISGylation of Interferon Regulatory Factor 3 (IRF3), a key transcription factor that orchestrates multiple IFN-mediated pathways. Concordantly, SCoV2-PLpro overexpression led to a significant reduction of IFN-I induction in response to treatment with poly(I:C), a double-stranded RNA analog that mimics viral stimulation. Of note, while similar results were obtained using SCoV1-PLpro, its ability to inhibit IFN-I signaling seemed reduced compared to SCoV2-PLpro in this setting. It is interesting to note that the authors identified multiple other specific cellular interactors of SCoV1-PLpro and SCoV2-PLpro, which could further be involved in their ability to modulate host immune responses. Moreover, given the complexity of immune regulation by ISG15, and its role in promoting USP18-mediated dampening of the IFN-I signaling in human,^5^ a complete understanding of its interplay with PLpro and its consequences on viral and host protein ISGylation should require further investigation.

**Fig. 1:**
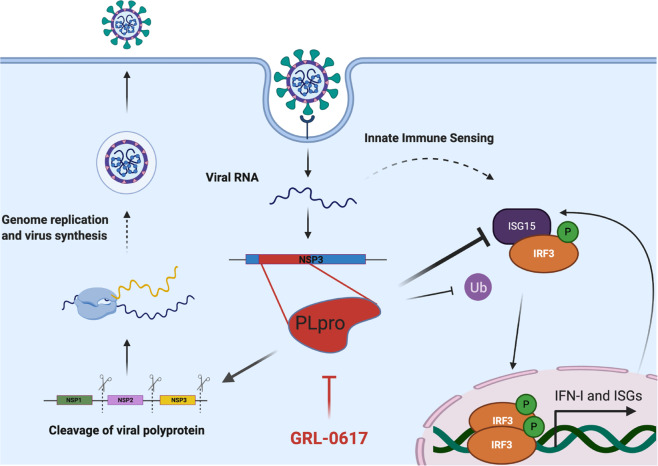
Dual role of SARS-CoV-2 protease PLpro in viral replication and inhibition of innate immune sensing. PLpro is required for the processing of SARS-CoV-2 polyprotein into mature sub-units to generate a functional replicase complex. Additionally, PLpro antagonizes the ISGylation of cellular proteins, including IRF3, leading the dysregulation of innate immune sensing

Viral proteases constitute one of the primary targets in drug discovery, as exemplified by several inhibitors successfully used in clinic against HIV and HCV proteases, among others. The dual role played by PLpro in promoting viral replication and inhibiting innate immune responses (Fig. 1) qualifies it further as an attractive target for drug development. The authors identified GRL-0617, a previously described SCoV1-PLpro inhibitor, as a potential drug candidate against SARS-CoV-2. Importantly, using similar assays to the ones performed in their study, the authors showed that GRL-0617 inhibits the deubiquitination and deISGylation activities of SCoV2-PLpro and restores the IFN-I response in SCoV2-PLpro-expressing cells stimulated with poly(I:C). Importantly, a 100 μM dose of GRL-0617 resulted in strong inhibition of viral replication and SARS-CoV-2 mediated cytotoxicity, paving the rationale to develop more potent inhibitors of SCoV2-PLpro.

Overall, this study sheds light on SARS-CoV2 PLpro and its role in the pathogenesis of COVID-19 by characterizing its deISGylation activity. It suggests that inhibition of PLpro would not only inhibits viral replication, but also rescue antiviral immunity, establishing PLpro as a particularly promising target for further drug development.
